# Integrating equity indicators for hospital reporting metrics

**DOI:** 10.1007/s43999-024-00046-w

**Published:** 2024-07-12

**Authors:** Aliya Allen-Valley, Shalu Bains, Karen Rai, Nirmal Summan, May Eleid, Emmalin Buajitti, Laura C. Rosella

**Affiliations:** 1 Institute for Better Health, Trillium Health Partners, Mississauga, ON Canada; 2 Trillium Health Partners, Mississauga, ON Canada; 3 Dalla Lana School of Public Health, University of Toronto, Toronto, ON Canada; 4 Department of Laboratory Medicine and Pathobiology, Temerty Faculty of Medicine, University of Toronto, Toronto, ON Canada

**Keywords:** Health equity, Inequity measurement, Health system, RII

## Abstract

Disparities in healthcare delivery and design are deeply-rooted within healthcare systems globally. Many researchers have developed methods to measure inequity; however, there currently exists no accepted measurement approach implemented consistently across health systems. We applied the model-based Relative Index of Inequality (RII) as a measure of inequity at one of Canada’s largest health systems, Trillium Health Partners, across two service types: planned and outpatient. Our RII estimates suggest that the lowest-SES individuals received planned and outpatient services at rates 2.4 times and 2.5 times lower than the highest-SES individuals, respectively. Across both service types, the largest disparity was for breast cancer screening, where patients from the lowest-SES neighbourhoods were 5.4 times less likely to use this service at THP. These findings further underscore the importance of consistently measuring and monitoring inequities to develop effective strategies to address the health needs of patients from lower SES neighbourhoods. The approach used within this study should be considered for widespread integration into health system reporting metrics.

**Supplementary Information:**

The online version contains supplementary material available at 10.1007/s43999-024-00046-w.

## Introduction

Inequities in the design and provision of healthcare services are deeply rooted within health systems around the world. Many disparities exist across a plethora of services and specializations and have been related to various sociodemographic factors [1–4]. Further, many equity-deserving groups experience limitations and additional barriers to health care access due to systemic racism and other barriers related to the social determinants of health (SDoH) [5, 6]. 

Health inequities are defined by the World Health Organization as “differences in health status or in the distribution of health resources between different population groups, arising from the social conditions in which people are born, grow, live, work, and age” [7]. In Canada, inequities related to service participation and delivery are well-demonstrated across various specializations, despite the presence of a single-payer publicly funded healthcare system. Nonetheless, across Canada, factors such as lower socioeconomic status (SES), geography, race, immigrant status, and ethnicity have been associated with adverse health outcomes such as increased mortality following a range of health events and outcomes such as Acute Myocardial Infarction [8], acute complications related to diabetes [9], and likelihood of no recent history of screening or later-stage diagnosis in cancer care [2, 10]. Similar patterns have also been found in regions and countries with similar healthcare structures, such as the United Kingdom [4] and Australia [11, 12], and in countries with differing heath care systems, like the United States. For example, a scoping review exploring disparities in cardiovascular care in England’s National Health Service (NHS) reported that 46% of included studies exploring SES found reduced access among groups with lower SES, and 49% of studies exploring differences related to ethnicity reported reduced access for racialized groups [4]. Findings across these studies further demonstrate the detrimental impact of these inequities on the health and well-being of the communities that these health systems seek to serve.

More recently, the importance of equity in health system performance has been articulated in The Quintuple Aim, which adds health equity as an integral component for the improvement of healthcare [6]. Moreover, many researchers have proposed different methods that can be used to measure and monitor inequities in attempts to close existing gaps [13–16]. However, despite this focus, no single approach for measuring inequities is used consistently across organizations providing health services.

Examining approaches to equity measurement across settings illustrates how much variation exists in the way health system inequities are measured and reported. For example, in the United Kingdom, Battersby et al. plotted incidence and procedure rates for non-small cell lung cancer against the weighted average of deprivation scores for primary care trusts (PCTs) – centres that coordinated health service provision [14]. In this case, the authors note that using weighted averages could have potentially led to bias in this study by masking deprivation levels within each PCT [14]. In the United States, Braveman et al. geocoded patient addresses to census tracts (CT), geographic areas that include approximately 4000–8000 people each, to analyze delayed or absent prenatal care, and used survey data to assign groups rankings based on socioeconomic factors, subsequently calculating the relative index of inequality (RII) [15]. However, in this case, the use of survey data was seen to be limiting to the ability to track data over time [15]. In Canada, Maddison et al. linked data from a population-based data-set on colorectal cancer care to clinical, demographic, and socioeconomic data from multiple databases and utilized the Horizontal Inequity Index (HI) as the measure of inequity, which explores where access is concentrated, and whether this is among advantaged or disadvantaged subgroups [16]. This approach focused on age, sex, income, and distance, and used a need-standardized approach, analyzing each of these indicators separately [16]. Finally, Waters et al. ranked populations by economic status quintiles in Ecuador [13]. This approach resulted in two different measures of inequity, as opposed to a single number.

These studies demonstrate the variation in inequity measurement in health systems and the need for an approach to measure inequity within these systems that results in a single indicator that can be easily tracked over time, thus supporting its integration into regular hospital reporting to address gaps in health service design and delivery. The approach proposed in this study used routinely collected neighbourhood-level SES characteristics based on widely available census data to measure inequity in service participation across two service types -- planned services (services requiring scheduling or hospital admission, excluding patients that entered the system via the emergency department or Labour and Delivery), and outpatient services (services often for diagnostic, initial specialty consultation, post surgery follow up or screening purposes) at one of Canada’s largest health systems by hospital volumes, Trillium Health Partners (THP).

THP is located in Peel Region, one of Ontario’s most diverse regions with a population of just under 1.5 million people in 2021 [17]. Of this population, 69% identified as racialized (compared to 34% of Ontarians and 27% of Canadians). Peel is also home to communities representing more than 450 ethnic and cultural origins per the 2021 Canadian Census [18]. Approximately 7.8% of the Region’s population was experiencing low income per the 2021 census [19]. The aim of this study was to understand differences in service use at THP based on neighbourhood-level characteristics of the diverse communities served within this healthcare organization, and to integrate inequity measures into THP’s regular reporting metrics; a novel step as THP is the first Canadian hospital to integrate inequity measurement into our reporting practices.

There are several metrics that have been used to measures inequities, including both relative and absolute measures. For this study, we used the Relative Index of Inequality (RII) - a unitless, regression-based measure -- to quantify differences in service utilization across both service types by SES. The RII was selected for as our primary measure because it considers visits across all quintiles and results in a single number, allowing for easier integration and interpretation for hospital performance reporting metrics, thus supporting our overarching objective: to report and apply this approach as a way for health systems to monitor health equity across the services they offer to support the design of services and outreach initiatives (i.e., awareness campaigns, community partnerships, and research endeavours) that aim to effectively identify and address existing gaps within the Peel Region.

## Methods

### Data sources and analysis

This study received Research Ethics Board (REB) approval from the University of Toronto (Protocol # 00044050). Health service volumes data of all THP visits for the 2021-22 fiscal year (FY 2021-22) were retrieved from THP’s decision-support team. These visits were reflective of all patient visits to the hospital, regardless of whether they fell within THP’s designated catchment area (North-West Missisauga, East Missisauga, South-West Mississauga, and South Etobicoke), though the large majority of analyzed visits were from individuals belonging to this catchment area, meaning that THP would be, in theory, the hospital that they would use for their services. This time period was selected to allow for the analysis of service use trends using the most recent data available, and data were collected and analyzed in alignment with THP’s fiscal years (April - March) to support the more seamless integration of findings into future data analysis methods used to inform service planning and performance monitoring cycles within the hospital system. Data retrieved by the project team included patients’ age, sex, postal code, service received, quintile information linked to dissemination area (DA), DA ID, Forward Sortation Area (FSA), and planned or outpatient care identifier.

Postal codes were matched to DAs using version 7D of Statistics Canada’s Postal Code Conversion File (PCCF+). DAs are geographic units containing 400–700 people [20]. We opted to conduct analysis at the DA-level as it is the smallest level at which census data is released providing a more granular SES estimate [20]. The match rate for this run of PCCF + was 83.98%.

Patient DAs were then linked to area-level material deprivation quintiles from the 2016 Ontario Marginalization Index (ON-MARG), and as such, each DA was provided with a material deprivation quintile of 1–5 [20]. Approximately 15,861 DAs were received overall; however, during this step, only DAs that fell within Ontario were able to be matched using this tool given that the ON-MARG solely provides insights related to Ontario geographic data. As such, DAs that fell outside of Ontario, or postal codes that were not able to be matched within ON-MARG (no match) were excluded from this analysis. In total, 7,124 unique DA’s were used for RII calculations.

Material deprivation scores are based on indicators related to factors such as income, education, housing quality and familial structure within a given area and were used as a proxy for SES. More specifically, this measure “refers to inability for individuals and communities to access and attain basic material needs” [20]. DAs assigned to Q1 included neighbourhoods with the highest SES, while DAs assigned to Q5 were comprised of neighbourhoods with the lowest SES.

### Planned and outpatient service definitions

Our study sought to calculate the RII specifically for services that required non-urgent referral into the system in order to assess whether THP services and outreach initiatives were effective and equitable in the provision of these services. As such, our analysis explored visits within two categories: Planned Services and Outpatient services.

Planned services were defined as those that required scheduling for hospital admission and excluded services for which patients entered the system through the emergency department (ED) or Labour and Delivery. Planned services include both Planned Surgeries and Planned Admits. Planned Surgeries include inpatient or day surgeries, and procedures requiring non-urgent referrals. Planned Admits were admissions categorized as elective based on Discharge Abstract Database (DAD) categories. Elective admits include “patients admitted for the scheduled treatment and/or assessment”, and “patients admitted from another facility for an Out-of-Hospital (OOH) intervention that was scheduled prior to the admission in the reporting OOH facility” [21]. 

Outpatient services were services that were often for diagnostic, initial specialty consultation, post surgery follow up or screening purposes and did not require an inpatient admission. Reoccurring scheduled outpatient visits (i.e., chemotherapy, dialysis, radiation) and pre-operative appointments were excluded. In total, 318,527 planned and outpatient visits with an associated postal code were included. Tables 1 and 2 detail the total number of visits per service type.

**Table 1:** Planned service (surgeries and planned admits) patient profiles, FY 2021–2022

Category	Value	
**Total N**	26,218	
**Top 3 Areas by Volume**		
Urology Surgery	35.20%	
Cataract Surgery	17.10%	
General Surgery	11.30%	
**Planned Admits average age (W&C, Oncology, Medicine)**	52.2	
**Planned Surgery average age**	63.1	
**Planned Admits + Surgery average age overall**	62	
**Planned surgery average age by subcategory**		
Cardiac Surgery	65.7	
General Surgery	58.7	
Gynecologic Oncology Surgery	59.1	
Neuro/Musculoskeletal Surgery	58.7	
Obstetrics and Gynecology Surgery	51.8	
Cataract Surgery	67.5	
Oral and Maxillofacial Surgery	36.4	
Orthopedic Surgery	60.8	
Otolaryngology Surgery	37.8	
Plastic Surgery	48.8	
Thoracic Surgery	62.7	
Urology Surgery	66.9	
Vascular Surgery	69.5	
**Gender, planned overall (admits + surgery)**		
Female	47%	
Male	53%	
Unknown	0.01%	

Caption: A table providing descriptive statistics pertaining to planned service patient visits in FY 2021–2022

Source: Author’s analysis of data from internal health system (EPIC) and Discharge Abstract Database (2021–2022). Notes: The fiscal year defined is based on April 2021- March 2022

**Table 2:** Outpatient service patient profiles, FY 2021–2022

Category	Value /% Value
Total *N*	292,309
**Top 3 Areas by Volume (Outpatient)**	
Neuro/Musculoskeletal Clinic	21%
Surgery Clinic	18%
Cardiac Clinic	17%
**Average Age**	
Overall	55.7
Ontario Breast Screening Program (OBSP)	59.8
Primary Care/Complex Continuing Care/Rehab Clinic	78.5
Surgery Clinic	56.2
Women’s And Children Clinic	25.5
Mental Health Clinic	41.4
Cardiac Clinic	66.3
Neuro/Musculoskeletal Clinic	55.1
Diagnostic Imaging Clinic	59.3
Medicine Clinic	53
**Gender, planned overall (excluding OBSP)**	
Female	55.9%
Male	44.07%
Unknown	0.03%

Caption: A table providing descriptive statistics pertaining to outpatient service patient visits in FY 2021–2022

Source: Author’s analysis of data from internal health system (EPIC) and Discharge Abstract Database (2021–2022). Notes: the fiscal year defined is based on April 2021-March 2022

Following these aforementioned steps, RII was calculated to explore differences in participation across planned and outpatient services.

### The relative index of inequality

RII was selected to measure inequity as opposed to other approaches (for example, subtracting or dividing Q5 from or by Q1) because RII considers individuals across all quintiles, as opposed to those solely in the highest and lowest groups. Furthermore, RII was seen to be favourable because it provides a single number that can be analyzed for each service and service type, respectively, over time; thus providing the opportunity to systematically analyze progress towards more equitable service use by integrating this measure into regular hospital reporting metrics. As such, the use of a single indicator for this purpose was seen to be ideal in allowing for the analysis of which services are facing greater inequities to inform respective service delivery and design processes. Moreover, use of the same indicator across each service provided allowed for easier uptake and comprehension among relevant teams and leads within this healthcare organization to ensure easier interpretation.

To calculate RII, material deprivation quintiles were converted to SES rank, which describes the proportion of the complete population which would be of lower SES compared to the midpoint of the quintile group. Specifically, Q1, or individuals in the highest SES were given a ranking of 0.90; Q2 was assigned a ranking of 0.70; Q3 received a ranking of 0.50 (the midpoint); Q4 a ranking of 0.30, and Q5, or individuals in the lowest SES, received a ranking of 0.10. As a sensitivity analysis, we re-ran the analysis using an alternative approach to ranking quintiles aligned with their share of the population. The results of this analysis are provided in the online Appendix 1.

Once each quintile was ranked, linear regression models were used to estimate RII for each specialization of interest. In this case, the independent variable was SES rank, while the dependent variable was the count of services performed for each specialization in question. RII was calculated from model parameters as $RII = \frac{intercept}{slope + intercept}$. This represents the ratio difference in predicted service utilization between the highest-SES (slope + intercept) and lowest-SES (intercept) individuals in the population. This method of RII calculation has been previously described elsewhere [22]. 

RII calculations were repeated for each service included in both planned and outpatient services. To provide insight into the overall level of inequity experienced across service types, we then calculated the overall weighted RII for planned and outpatient services, respectively, to account for the volume by subcategory under planned or outpatient services.

RII should be interpreted as the model-based difference between the hypothetically lowest-SES individual in a population, compared to the hypothetically highest-SES individual. For each calculated RII, a value greater than one (RII > 1) indicated that those neighbourhoods with the lowest SES were using a given service at lower rates than those from higher SES neighbourhoods. Inversely, an RII less than one (RII < 1) indicated that those from lower SES neighbourhoods were using a given service at a higher rate than those from higher SES neighbourhoods. An RII of one (RII = 1) indicated that there was no difference in use of a given service based on neighbourhood SES. As RII are model-based and utilize information from across socioeconomic strata (i.e. quintiles 1, 2, 3. 4, and 5), estimates may differ from traditional pairwise comparisons of highest- and lowest-SES neighbourhoods (for example, a Q5/Q1 risk ratio). Weighted planned and outpatient services are based on volume and RII value per subcategory, which are then accounted for each category overall (planned and outpatient, respectively).

## Results

### Patient profiles

Tables 1 and 2 provide descriptive statistics of all patients receiving planned and outpatient services at Trillium Health Partners, respectively, during the 2021–2022 fiscal year. Among planned services, the largest volume of patients was seen among urology surgery patients (35.2%), cataract surgery (17.1%) and general surgery (11.3%).

Among outpatient services, the largest volumes were seen for Neuro/Musculoskeletal (Neuro/MSK) clinic (21%), surgery clinic (18%), and cardiac clinic (17%) visits. Moreover, a much greater volume of outpatient services was performed as compared to planned services, with volumes of 292,309 and 26,218, respectively. For planned services, more services were performed on male patients (53%) than female patients (47%). However, for outpatient services, the inverse was true; 55.9% of services were performed on female patients, and 44.07% of services were performed on male patients. The average age across all outpatient services (55.7 years) was lower than for planned services (62.0 years).

Volumes varied across planned and outpatient services by quintile (Table 3). Across all specializations, the service at THP with the largest percentage of patients residing in neighbourhoods with the highest SES (Q1) was the Ontario Breast Screening Program (OBSP) (28.49%). Contrarily, the specialization with the largest percentage of visits from patients residing in neighbourhoods with the lowest SES (Q5) was observed among cancer planned admits (26.74%). An overview of participation volumes for all planned and outpatient services stratified by SES quintile is available in Table 3.

**Table 3:** Planned (admits and surgery) and outpatient service participation volumes by quintile, FY 2021–2022

	Quintile					
Service Type	Q1	Q2	Q3	Q4	Q5	Total
**Planned Admits**						
Cancer Admits	32	35	36	23	46	172
Medicine Admits	67	81	114	83	77	422
Women & Children Admits	100	142	132	89	65	528
**Planned Surgery**						
Cardiac Surgery	120	117	111	76	47	471
General Surgery	621	795	734	505	317	2972
Gynecologic Oncology Surgery	136	113	145	95	76	565
Neuro/Musculoskeletal Surgery	269	250	234	179	130	1062
Obstetric and Gynecology Surgery	243	275	253	178	107	1056
Cataract Surgery	918	1089	1100	851	519	4477
Oral and Maxillofacial Surgery	81	79	53	50	40	303
Orthopedic Surgery	588	672	605	396	277	2538
Otolaryngology Surgery	238	252	233	153	108	984
Plastic Surgery	113	117	114	80	60	484
Thoracic Surgery	75	81	61	46	35	298
Urology Surgery	2128	2491	2275	1407	932	9233
Vascular Surgery	126	166	164	102	95	653
**Outpatient**						
Ontario Breast Screening Program	3925	3778	3149	1801	1124	13,777
Primary Care/Complex Continuing Care /Rehab Clinic	2117	2225	1890	1477	871	8580
Surgery Clinic	12,600	13,574	12,752	8463	5563	52,952
Women & Children Clinic	6645	7210	7452	4850	3116	29,273
Mental Health Clinic	129	96	146	87	52	510
Cardiac Clinic	10,820	13,134	13,051	8180	5324	50,509
Neuro /Musculoskeletal Clinic	13,605	15,926	14,875	9988	6920	61,314
Diagnostic Imaging Clinic	6668	7128	6814	4949	3340	28,899
Medicine Clinic	8904	11,221	11,801	8170	6399	46,495

Planned Admits, Planned and Outpatient Participation Rates by Quintile

Caption: Table providing planned and outpatient service volumes by quintile, fiscal year 2021–2022

Source: Author’s analysis of data from internal health system (EPIC) dataset, Discharge Abstract Database, and 2016 Ontario Marginalization Index. Postal code data converted to Dissemination Areas using Stats Canada’s Postal Code Conversion File (PCCF). Notes: the fiscal year defined is based on April 2021-March 2022

**Fig. 1:**
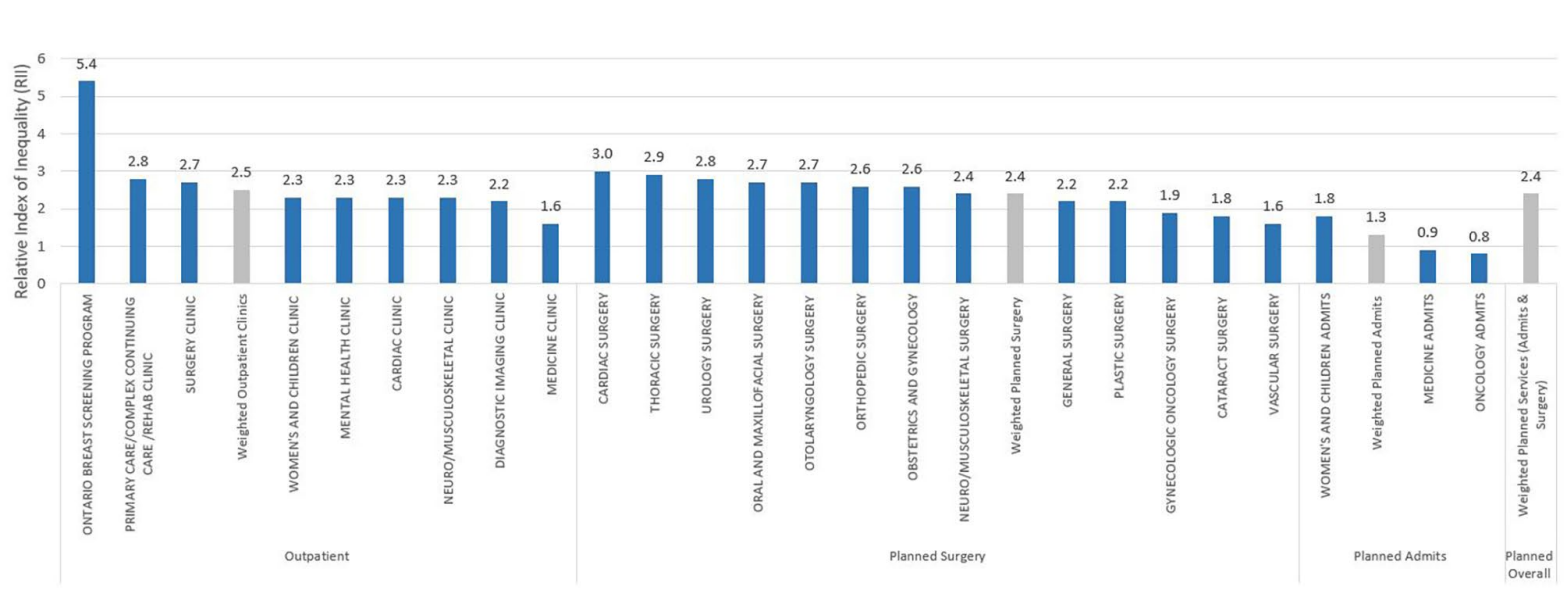
presents the RII for each planned and outpatient service at THP (Fig. 1). Further details regarding RII findings across both planned and outpatient services are outlined below

Figure 1 RII Values for Planned Admits, Planned, and Outpatient Services (FY 2021–2022). A bar graph showcasing the Relative Index of Inequality (RII) for outpatient and planned (planned surgery and planned admits) visits for FY 2021–2022. The weighted average for each category is also provided. Source: Author’s analysis of data from internal health system (EPIC) dataset, Discharge Abstract Database, and 2016 Ontario Marginalization Index. Postal code data converted to Dissemination Areas using Stats Canada’s Postal Code Conversion File (PCCF). Notes: the fiscal year defined is based on April 2021-March 2022.

### Planned surgeries

Planned services included those that required scheduling for hospital admission and excluded services for which patients entered the system through the emergency department (ED) or Labour and Delivery. This category includes planned surgeries and other planned admits into the hospital.

Across all planned inpatient and day surgeries, the weighted RII was 2.4, suggesting that the lowest-SES patients are expected to be 2.4 times less likely to receive a planned surgery service, compared to the highest-SES patients. The largest disparities were observed for cardiac surgery and thoracic surgery, for which low-SES patients were an estimated 3.0 times less likely to receive cardiac surgery, and an estimated 2.9 times less likely to receive thoracic surgery. An RII greater than 1 (indicating SES disparity) was observed for all services, with the smallest RII (1.6) seen for vascular surgery.

### Planned non-surgical admits

Among other planned admits, the lowest-SES individuals were expected to be 1.8 times less likely to receive women’s and children’s services, and were slightly more likely to come in for medicine (RII = 0.9) and oncology (RII = 0.8) admits compared to the highest-SES individuals. Across all planned admits, the lowest-SES patients were 1.3 times less likely to receive these services, as demonstrated by the weighted average.

### Outpatient services

Outpatient services were often for diagnostic, initial specialty consultation, post surgery follow up or screening purposes, and reoccurring scheduled outpatient visits like chemotherapy, dialysis, radiation, and pre-operative appointments were excluded. The weighted RII across all outpatient services was 2.5, indicating that we expect lowest-SES patients to be 2.5 times less likely to receive these services than high-SES patients. Across both service types (planned and outpatient), the greatest disparity within our health system was observed for the Ontario Breast Screening Program (OBSP), where low-SES patients were 5.4 times less likely to participate in this service at THP compared to high-SES patients. The smallest disparity was noted among medicine outpatient services; however, in this case, individuals from low SES neighbourhoods were still an estimated 1.6 times less likely to receive these services than individuals from high SES neighbourhoods. The results of our sensitivity analysis demonstrated that an alternate approach of assigning rankings did not significantly our findings. Appendix 1 highlights the estimated RII for each service type using both approaches.

## Discussion

Our findings demonstrate clear differences in planned and outpatient service participation based on neighbourhood-deprivation levels, such that in almost all cases, individuals from lower-SES neighbourhoods were less likely to receive planned and outpatient services within one of Canada’s largest health systems. These findings align with previous research conducted in Canada and other countries, where research has demonstrated similar trends across a multiplicity of service types. In many cases, these differences were also in relation to geographic or locational differences [1, 3, 9, 14, 16]. In addition to geographic differences, other Canadian, U.K., and U.S.-based studies have also found differences related to sociodemographic factors such as ethnicity, [2, 3] age and sex [16], and income or SES [4, 23]. For this reason, many studies, including our own, have highlighted the need to measure inequity within health service provision in order to address it over time [13–15, 24]. While many methods have been proposed to measure inequity within hospital systems, we have proposed use of the RII due to its ability to produce a single number that can be (a) tracked over time, and (b) integrated into health system performance reporting metrics for continuous analysis. Moreover, the ability of this metric to consider differences across all quintiles, as opposed to solely the least- and most-deprived groups, is of interest because it allows for the consideration of all patients receiving services. While there are more complex measures that offer methodological advantages, the logistical challenges of reporting and presenting these measures interferes with the ability to integrate into regular reporting, thereby limiting the attention and accountability to continuously monitoring inequities in health systems.

It is important to note that we are continuously evolving our definitions of planned and outpatient services in collaboration with clinical partners to best align with THP’s context. As other health systems may adopt and utilize this approach for inequity measurement, it is important to ensure that the categories and definitions used within their contexts align with the specific aims, needs, and objectives of their organization(s). Future iterations of this analysis at THP will include an updated methodology to separate ambulatory visits attached to unplanned inpatient admissions.

While disparities were demonstrated across almost all services, the most pronounced differences were observed among the Ontario Breast Cancer Screening Program (OBSP) within our system. This is of significant concern as cancer screening has been found to lead to better health outcomes, including improved survival rates [10, 25]. These findings are also aligned with previous work. Lofters. et al. found that over one-fifth of individuals living in Ontario that were eligible for breast, cervical and colorectal cancer screening had no history of screening in the past five years or longer, and individuals who did not participate in screening programs were also more likely resided in lower-income neighbourhoods [10]. Our current findings may also be reflective of larger disparities in accessibility or cultural appropriateness of this breast screening services within our system. Further research, perhaps of a qualitative nature, will support the ability of our system to further understand the causes of these inequities, as well as trends in need and utilisation across different communities and how this may vary in different settings. It is also important to note that while an RII of 1 may indicate *equal* service use between high- and low-SES settings, an RII of 1 may not necessarily be reflective of *equitable* use across different communities, as we know that need across different communities and settings may vary based on a multitude of factors. Nonetheless, the recognition of extreme disparities in service use across different service types between those from lower- and higher- SES neighbourhoods may be reflective of further underlying inequities between these communities; the nature and extent of which must be explored and addressed to truly move towards more equitable health outcomes within and across our communities. Understanding the magnitude of these differences in need, as well as the steps that must be taken to address the root causes of these differences if needed, are truly required to ensure that the communities that we serve have access to the services they need, both within and outside of the hospital system. Taken together, the present findings and findings from previous studies further emphasize the need for improved service planning and delivery processes to consider how inequities in service use can be measured within hospital systems to design solutions that prevent adverse health outcomes. In this study, we chose to use the RII, a unitless single measure that describes relative inequalities that is easily interpreted. Other relative metrics, such as the concentration index, similarly use ranks to estimate the degree of inequality according to the twice the area between the concentration curve and the line of equality. In addition, we chose to report a relative measure given the preferences for interpretation in our setting; however, we provide the absolute differences as additional data and the descriptive rates by SES quintile since both relative and absolute measures provide a comprehensive understanding of inequities. Future research that compares the usability of such measures, including their ability to be interpreted for health system monitoring, will continue to be important in informing the choice of indicator.

There are some limitations of our study, which are important to note. First, while DAs are nationally utilized in smaller geographical units and are recommended for use when area-based data is used as a proxy for SES [20], an area-based approach to measuring SES may not accurately capture individual-level SES for all patients. Descriptions of lower or higher socioeconomic position apply solely to neighbourhoods and not necessarily to all individuals living within them. However, we chose this approach because individual-level SES data is not routinely collected or available in Canadian health systems and therefore, relying only on individual measures would prevent the opportunity for routine reporting. Postal code and census data are always available and thus can ensure regular and routine reporting. Secondly, RII provides a point-in-time snapshot of inequality within a given area and may not maintain its validity following shifts in the socioeconomic distribution of a given community that may occur over time. Another potential limitation is that this analysis was only able to capture data from patients with a valid health-card number; as such, individuals without a health card were excluded from analysis. Finally, while findings demonstrate differences in service participation, they do not account for potential differences in need within a given neighbourhood (i.e., whether there are increased health conditions within given quintiles that may account for increased use). While we believe that the RII, as currently presented, is an extremely useful tool to monitor the degree of inequities that exist and a starting point for subsequent engagement and analysis that reveal the differences in service use that exist within a given health system, we recognize that subsequent steps and measures will follow to understand why the degree of difference in service use being observed exists in order to address recognized gaps meaningfully. For example, future approaches to calculating the RII may consider including an analysis of differences in expected need across neighbourhoods, which may, in some cases, be reflective of underlying inequities within different settings to understand how needs relate to observed differences in service use [14]. The needs assessment should consider specific needs dimensions for the healthcare service being measured and unique to the local context to be most effective.

The RII, as presented, can demonstrate the level of inequity in service use between neighbourhoods by using routinely collected data that can be replicated across service areas. However, information pertaining to *why* these gaps exist, accounting for existing levels of need within different jurisdictions or barriers and facilitators to service use across different neighbourhoods, must be explained through additional approaches, including quantitative and qualitative methods. Building upon the RII and triangulating data collection approaches to further detail the drivers of existing gaps in service use will support the development of more meaningful solutions to existing gaps to improve healthcare outcomes for communities. Nonetheless, the RII has demonstrative value as an addition to our health system performance metrics, as it has provided us with insight into which communities should be engaged for future research to understand differences in service use. The next steps include qualitative studies with people living in neighbourhoods experiencing lower service use and comparisons on various need dimensions, which will elucidate specific community needs and barriers. Conducting qualitative studies and co-design sessions to remove revealed barriers are key next steps we plan to undertake to sharpen our ability to understand and address recognized gaps in service use. Finally, because we are using our measure as a descriptive indicator, we did not further adjust for other factors [26, 27]. However, further analysis that allows for well-specified-causal questions related to socioeconomic factors can incorporate further adjustments and inform interventions as appropriate. [14].

## Conclusion

Our study has demonstrated an approach that can be used to measure and monitor health inequities in health systems using routinely collected data. Our findings reveal that large differences exist in use of planned and outpatient hospital services, specifically in relation to neighbourhood deprivation. These findings demonstrate a need to identify barriers and facilitators to hospital service use within our system, specifically for those in lower SES neighbourhoods. Our findings also demonstrate the need to develop standardized approaches to measure inequities in healthcare that are easily adopted and scalable in order to develop impactful solutions and to improve use of services across the communities that we seek to serve.

The approach presented in our study can be considered to measure inequity for healthcare systems and institutions to measure inequity regularly, in their performance reporting metrics to continue progress towards addressing these inequities and truly become population health focused organizations.

### Electronic supplementary material

Below is the link to the electronic supplementary material.

Supplementary Material 1
