# Supplementary material for: Integrating equity indicators for hospital reporting metrics
Source: Res Health Serv Reg. 2024 Jul 12;3:10. doi: 10.1007/s43999-024-00046-w (PMC11281735; doi:10.1007/s43999-024-00046-w)
Supplement: Supplementary file 1 — Supplementary Material 1 [file 43999_2024_46_MOESM1_ESM.pdf]

Supplementary Information

The authors would like to note that in addition to providing rankings to each material deprivation quintile ranging from .9 in Q1 to .1 for those in Q5 to reflect the share of the population purported to be experiencing lower socioeconomic status than those in the midpoint of each respective quintile. As a sensitivity analysis, we also used a population-based approach, which provided each quintile with a ranking proportionate to their share of the population to explore whether this would significantly change any of our reported findings. This approach only slightly changed the ranking of each quintile, such that those in Q1 were provided a rank of .88; those in Q2 a ranking of .66 (previous ranking .7); those in Q3, the midpoint of the population, were provided a ranking of .46 (previous ranking .5), and those in Q4 were provided a ranking of .28 (previous ranking .3). Using this approach, the weighted RII for each service remained relatively unchanged (weighted outpatient RII = 2.3; weighted planned surgery RII = 2.4; weighted planned services RII = 2.3). The largest change was observed for the Ontario Breast Screening Program, where the RII changed from a previous 5.4 to 5.1. Appendix 1 provides a chart highlighting the changes in RII values between each service type using this approach. Nonetheless, these new findings continue to reflect large discrepancies in service use between those in high- and low-socioeconomic status neighbourhoods, such that those from low-SES neighbourhoods were far less likely to use each service. As such, our key findings remain unchanged.

Appendix 1

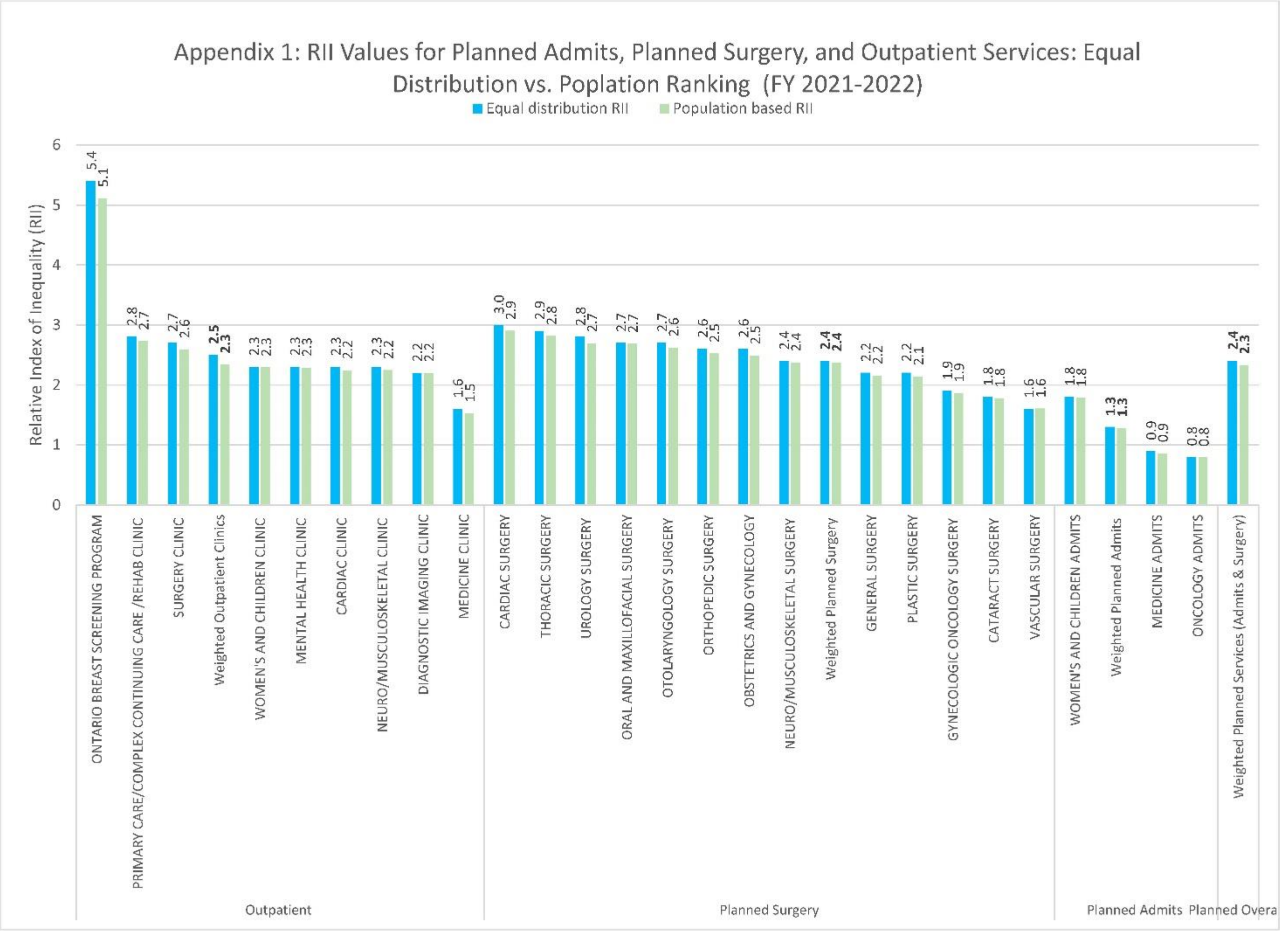

Appendix 1 A bar graph showcasing the Relative Index of Inequality (RII) for outpatient and planned (planned surgery and planned admits) visits for FY 2021-2022 using both an equal distribution and a population-based ranking approach, demonstrating minimal differences in RII estimates across each service type
